# Association between dietary selenium intake and the prevalence of hypertension: results from the National Health and Nutrition Examination Survey 2003–2018

**DOI:** 10.3389/fimmu.2024.1338745

**Published:** 2024-01-16

**Authors:** Yilin Wu, Zongliang Yu

**Affiliations:** Department of Cardiology, Affiliated Kunshan Hospital of Jiangsu University, Kunshan, Jiangsu, China

**Keywords:** selenium, hypertension, NHANES, cross-sectional study, RCS

## Abstract

**Aim:**

The epidemiological evidence regarding the impact of dietary selenium intake on hypertension continues to be a subject of controversy. Our objective is to examine the correlation between dietary selenium intake and the prevalence of hypertension within a substantial and diverse population in the United States.

**Methods:**

We carried out a cross-sectional study using data from the National Health and Nutrition Examination Survey (NHANES) to assess the association between dietary selenium intake and hypertension prevalence. Weight logistic regression analysis and smooth curve fitting were utilized to explore potential linear relationships. Subgroup analysis was further employed to investigate potential differences in this relationship across populations and assess potential synergies.

**Results:**

The study included 32,928 individuals, and the average dietary selenium intake was 1.12 ± 0.53 μg. The prevalence rate of hypertension was 36.55% overall and decreased with the higher dietary selenium intake quartiles (quartiles 1, 40.25%; quartiles 2, 37.71%; quartiles 3, 36.04%, quartiles 4, 32.23%, p < 0.001). Each quartile increase in dietary selenium intake associated with 11% decreased the likelihood of prevalence of hypertension [OR = 0.89; 95% CI: 0.80–1.00; p = 0.0425]. Subgroup analyses revealed that there was no significant correlation between gender, age, body mass index, smoking status, alcohol consumption, and diabetes mellitus in relation to the association between dietary selenium intake and the prevalence of hypertension.

**Conclusion:**

The prevalence of hypertension in adults was found to be linearly and negatively correlated with dietary selenium intake. In order to improve the prevention and treatment of hypertension, greater emphasis should be placed on selenium consumption.

## Introduction

Hypertension, a progressive and damaging cardiovascular disease characterized by persistent elevation of arterial blood pressure, is the highest death toll worldwide, accounting for roughly 14% of all deaths (1). The World Health Organization reports a yearly rise in the incidence of hypertension, which influences more than 1.3 billion people globally (2), and the age at which it starts tends to be younger. Dietary factors, exceptionally high salt, low potassium diets, and excessive alcohol intake are significant contributors to the development of hypertension (3). To prevent the start and progression of hypertension, a deeper comprehension of the connection between dietary practices and hypertension risk is necessary.

Selenium is a necessary trace element that is a part of selenoproteins, which have a significant physiological function in the body. Selenoproteins contain the most prominent selenium compound, selenocysteine, an antioxidant and redox regulator that protects cells from oxidative stress and free radical damage (4). It is reported that the immune system, oxidative stress, and the inflammatory response are all involved in the initiation and progression of hypertension (5). These factors are also intimately linked to the consequences of hypertension, such as cardiac damage, hemorrhagic stroke, and renal injury (6).

Remarkably, prior epidemiologic research has concentrated chiefly on the value of blood selenium levels rather than emphasizing selenium intake. Therefore, we used data from the National Health and Nutrition Examination Survey (NHANES) from 2003–2018 to explore the association between dietary selenium intake and the prevalence of hypertension.

## Methods

### Survey description

NHANES is a population-based survey that the National Center for Health Statistics (NCHS) employs to collect data on the health and diets of Americans. In order to obtain exceptionally precise results, the survey utilizes a stratified, multistage probability cluster sampling methodology over a two-year period. All survey participants furnished written informed consent, and the Research Ethics Review Board of NCHS granted approval for each NHANES study protocol. The design and data of the NHANES study are described in detail at www.cdc.gov/nchs/nhanes/.

### Study population

**Figure 1:**
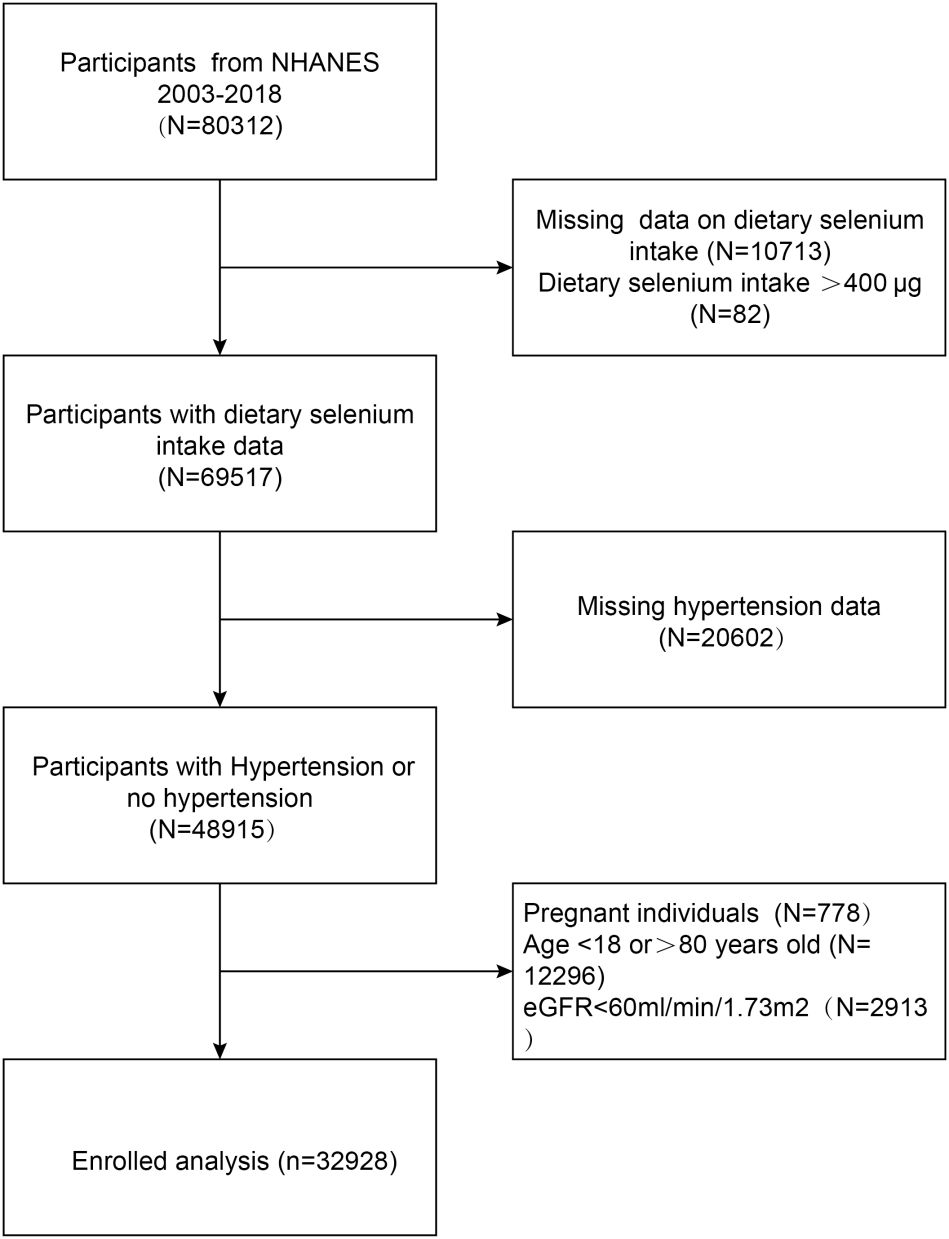
Flowchart of the sample selection from the National Health and Nutrition Examination Survey (NHANES) 2003–2018.

The study delved into exploring the connection between dietary selenium intake and the prevalence of hypertension, utilizing comprehensive data gathered from the National Health and Nutrition Examination Survey (NHANES) conducted between 2003 and 2018. The subject exclusion criteria were as follows: 1) individuals who are under the age of 18 or over the age of 80; 2) those who have an estimated glomerular filtration rate (eGFR) of less than 60 ml/min/1.73m^2^, the eGFR is computed using the CKD-EPI equation, which was introduced in 2009 and offers enhanced precision and dependability; 3) expectant women; 4) participants who do not have information regarding their dietary selenium intake and have a daily selenium intake exceeding 400 μg; 5) those who lack comprehensive hypertension data. We ultimately incorporated 32,928 participants into our analysis (**Figure 1**).

### Exposure and outcomes

Dietary selenium intake was an independent variable. An automated multiple-pass method was used to collect dietary data for large-scale national surveys such as NHANES, providing a highly efficient and accurate collection process. The experienced dietary interviewer conducted two 24-hour dietary recalls. The first survey was conducted face-to-face and the second by telephone 10 days later. According to USDA’s Dietary Research Food and Nutrition Database, the average dietary selenium intake was calculated from two recalls using nutrient values. This study limited selenium intake to 400μg of selenium per day, the upper tolerable limit for adults.

The outcome variable was hypertension. Three consecutive blood pressure readings are taken after the participant has rested quietly for 5 minutes and determined their maximum inflation level (MIL). It is possible to take a fourth measurement if the previous measurements were interrupted or incomplete. The Mobile Examination Center (MEC) took all blood pressure measurements (systolic and diastolic). hypertension was recognized as the following:1) at least 140 mmHg of mean systolic blood pressure (SBP); 2) at least 90 mmHg of mean diastolic blood pressure (DBP); 3) self-reported hypertension; 4) utilizing antihypertensive medications. Based on a guideline from the International Society of Hypertension, the 140/90 mmHg threshold was established.

### Covariates

We obtained demographic information from the questionnaire, household poverty-to-income ratio (PIR), smoking, alcohol consumption, and diabetes disease history from the health questionnaire. Blood samples were obtained in order to analyze the blood biochemical indices after a minimum of 8 hours of fasting during the overnight period. The serological indices examined in this study encompassed HbA1c(%), serum creatinine(Scr, mmol/L), serum uric acid(UC, ummol/L), total cholesterol(TC, mmol/L), triglycerides(TG, mmol/L), high-density lipoprotein cholesterol(HDL-C, mmol/L), low-density lipoprotein cholesterol (LDL-c, mmol/L), serum urea nitrogen (BUN, mmol/L), albumin (ALB, mmol/L), glutamic-pyruvic transaminase (ALT, mmol/L), and glutamic oxalacetic transaminase (AST, mmol/L). Systolic blood pressure (SBP) and diastolic blood pressure (DBP) measurements were acquired during the clinical examination.

The Body Mass Index (BMI) was classified into three categories: <25, 25-29.9, and ≥30 kg/m2, representing individuals with normal weight, overweight, and obesity, respectively, among the study participants. Consuming a minimum of 12 units of alcoholic drinks annually may be classified as alcohol consumption, whereas engaging in smoking with a lifetime consumption of at least 100 cigarettes can be categorized as tobacco use.

### Statistical analysis

R (version 3.4.3, http://www.R-project.org) and EmpowerStats software (http://www.empowerstats.com) were used for all analyses. Continuous variables are presented as mean ± standard deviation (SD), and categorical variables are presented as percentages. Linear regression models were used for continuous variables, and chi-square tests were used for categorical variables to analyze baseline characteristics. We classified dietary selenium intake into four categories. A multivariate logistic regression model was used to examine the independent association between dietary selenium intake and the prevalence of hypertension after adjusting for potential confounders. Model 1 did not include covariates, Model 2 adjusted for age, gender, and race, and Model 3 added education, PIR, BMI, alcohol, smoking, diabetes, HbA1c, Scr, UC, TC, TG, HDL-c, LDL-c, HB, ALB, ALT, AST, SBP, and DBP as covariates to Model 2. A value of p < 0.05 was considered statistically significant.

## Results

### Baseline characteristics of participants

The study enrolled a cohort of 32,928 individuals, of which 51.03% were male and 48.97% were female. The average age of the participants was 45.12 ± 17.42 years. The prevalence of hypertension was 36.55% and decreased with increasing quartiles of dietary selenium intake. The prevalence of hypertension was 40.25%, 37.71%, 36.04%, and 32.23% in quartiles 1, 2, 3, and 4, respectively. Subjects with increased dietary selenium intake were male, smokers, alcohol drinkers, non-Hispanic White, educated to college level and above, younger, with lower BMI, ALB, HbA1c, HDL-C, TC, SBP, and higher Hb, ALT, AST, BUN, TG, CR, UC, DBP (all p<0.05). The differences in LDL-C between quartiles were not statistically significant (p > 0.05). **Table 1** lists the clinical and biochemical characteristics of the participants according to the dietary selenium intake quartiles.

**Table 1:** Baseline characteristics of participants.

Variables	Overall	Q1	Q2	Q3	Q4	*p*-Value
		N=8192	N=8160	N=8329	N=8247	
Hypertension,n (%)	12034 (36.55%)	3297 (40.25%)	3077 (37.71%)	3002 (36.04%)	2658 (32.23%)	<0.001
Age,years	45.12 ± 17.42	47.01 ± 18.24	46.48 ± 17.73	44.91 ± 17.12	42.10 ± 16.10	<0.001
Race,n(%)						<0.001
Mexican American	5765 (17.51%)	1411 (17.22%)	1412 (17.30%)	1439 (17.28%)	1503 (18.22%)	
Other Hispanic	3063 (9.30%)	842 (10.28%)	749 (9.18%)	762 (9.15%)	710 (8.61%)	
Non-Hispanic White	13593 (41.28%)	3203 (39.10%)	3447 (42.24%)	3573 (42.90%)	3370 (40.86%)	
Non-Hispanic Black	7173 (21.78%)	2043 (24.94%)	1758 (21.54%)	1685 (20.23%)	1687 (20.46%)	
Other	3334 (10.13%)	693 (8.46%)	794 (9.73%)	870 (10.45%)	977 (11.85%)	
Gender,n(%)						<0.001
male	16802 (51.03%)	2492 (30.42%)	3419 (41.90%)	4683 (56.23%)	6208 (75.28%)	
Female	16126 (48.97%)	5700 (69.58%)	4741 (58.10%)	3646 (43.77%)	2039 (24.72%)	
Education,n (%)						<0.001
Less than 9^th^ grade	3039 (9.92%)	1055 (13.94%)	801 (10.51%)	649 (8.35%)	534 (6.97%)	
9-11^th^ grade	4298 (14.03%)	1179 (15.58%)	1057 (13.87%)	1047 (13.47%)	1015 (13.24%)	
High school graduate	7063 (23.06%)	1860 (24.58%)	1760 (23.09%)	1672 (21.51%)	1771 (23.11%)	
Some college or AA degree	9205 (30.05%)	2158 (28.51%)	2290 (30.04%)	2403 (30.91%)	2354 (30.71%)	
College graduate or above	7024 (22.93%)	1316 (17.39%)	1714 (22.49%)	2003 (25.77%)	1991 (25.98%)	
Alcohol use, n (%)	19077 (72.51%)	4102 (63.69%)	4523 (69.29%)	5121 (75.99%)	5331 (80.76%)	<0.001
Smoking,n (%)	14019 (44.69%)	3360 (43.23%)	3387 (43.42%)	3539 (44.50%)	3733 (47.61%)	<0.001
Poverty-income ratio	2.51 ± 1.64	2.25 ± 1.57	2.51 ± 1.63	2.64 ± 1.66	2.64 ± 1.66	<0.001
BMI, kg/m2	29.18 ± 6.87	29.38 ± 6.94	29.21 ± 6.82	29.40 ± 6.93	28.73 ± 6.77	<0.001
Diabetes, n (%)	19077 (72.51%)	1008 (12.58%)	977 (12.22%)	895 (10.99%)	727 (8.99%)	<0.001
HbA1c, %	5.69 ± 1.06	5.72 ± 1.08	5.70 ± 1.05	5.70 ± 1.08	5.63 ± 1.02	<0.001
UC, ummol/L	320.90 ± 82.43	307.29 ± 81.66	313.89 ± 82.42	325.42 ± 81.69	336.45 ± 80.95	<0.001
HDL-c, mmol/L	1.35 ± 0.40	1.39 ± 0.42	1.37 ± 0.40	1.35 ± 0.39	1.31 ± 0.39	<0.001
TG, mmol/L	1.44 ± 1.33	1.38 ± 1.01	1.44 ± 1.33	1.44 ± 1.27	1.50 ± 1.61	0.001
TC, mmol/L	4.96 ± 1.08	4.98 ± 1.10	4.97 ± 1.09	4.95 ± 1.07	4.92 ± 1.07	0.002
LDL-c, mmol/L	2.92 ± 0.91	2.93 ± 0.95	2.93 ± 0.90	2.92 ± 0.89	2.90 ± 0.91	0.438
Scr, mmol/L	75.63 ± 18.65	72.05 ± 19.21	73.89 ± 17.82	76.63 ± 18.06	79.80 ± 18.57	<0.001
BUN, mmol/L	4.53 ± 1.61	4.18 ± 1.57	4.48 ± 1.62	4.65 ± 1.58	4.80 ± 1.59	<0.001
ALB,mmol/L	42.56 ± 3.36	42.04 ± 3.34	42.29 ± 3.33	42.68 ± 3.33	43.20 ± 3.34	<0.001
ALT, mmol/L	25.63 ± 21.19	23.50 ± 22.87	24.79 ± 23.68	26.13 ± 18.30	28.00 ± 19.28	<0.001
AST, mmol/L	25.53 ± 17.73	24.97 ± 21.45	25.09 ± 15.67	25.50 ± 14.42	26.53 ± 18.58	<0.001
SBP, mmHg	122.14 ± 17.14	122.69 ± 18.75	122.36 ± 17.73	121.93 ± 16.51	121.61 ± 15.37	<0.001
DBP,mmHg	70.17 ± 12.19	69.45 ± 12.32	69.92 ± 12.14	70.34 ± 12.18	70.97 ± 12.07	<0.001
Selenium (100 μg)	1.12 ± 0.53	0.56 ± 0.15	0.90 ± 0.08	1.20 ± 0.10	1.84 ± 0.44	<0.001

Continuous variables are presented as the mean and 95% confidence interval, categorical variables are presented as the proportion and 95% confidence interval. PIR, household poverty-to-income ratio; BMI, Body Mass Index; WC, waist circumference; HbA1c, glycated hemoglobin; UC, serum uric acid; UC, serum uric acid; TG, triglyceride; TC, total cholesterol; TC, total cholesterol; LDL-c, low-density lipoprotein cholesterol; HDL-c, high-density lipoprotein cholesterol; CR, serum uric acid; BUN, serum urea nitrogen; ALB, glutamic-pyruvic transaminase; ALT,glutamate pyruvic transaminase; AST, glutamic oxalacetic transaminase,SBP, systolic blood pressure; DBP, diastolic blood pressure.

### The relation between dietary selenium intake and prevalence of hypertension

**Figure 2:**
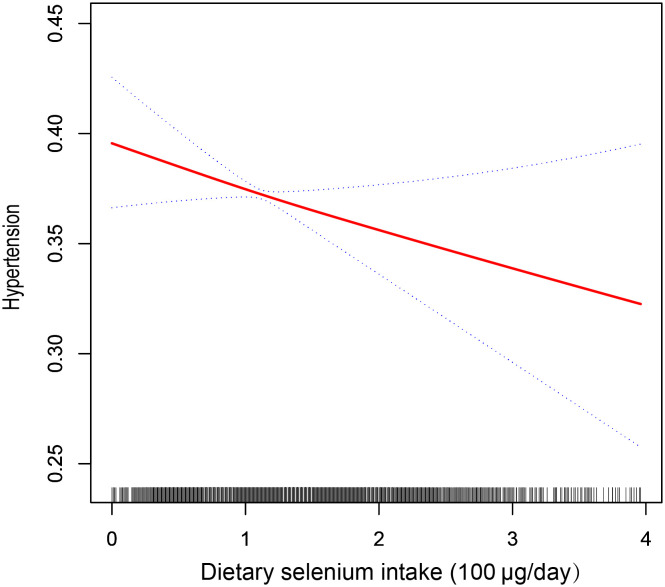
The relationship between dietary selenium intake and hypertension. The solid line indicates the estimated prevalence of hypertension, and the dotted lines represent a 95% confidence interval from the fit. Adjusted for gender, age, race, education, PIR, BMI, WC, alcohol, smoking, diabetes, HbA1c, Scr, UC, TC, TG, HDL-C, LDL-C, HB, ALB, ALT, AST, SBP, and DBP.

In the multivariate logistic regression models, an inverse correlation between dietary selenium consumption and hypertension prevalence. Both the crude (OR=0.97; 95% CI: 0.92-1.03; p =0.3202) and fully adjusted models (OR=0.89; 95% CI: 0.80-1.00, p=0.0425) indicated that higher dietary selenium intake was associated with a lower prevalence of hypertension. The fully adjusted model revealed an 11% reduction in the probability of hypertension prevalence for every unit increase in dietary selenium intake. Sensitivity analyses using dietary selenium intake as a categorical variable (quartiles) supported these findings, with participants in quartile 4 (139 – 400μg/day) showing a 20% reduction in hypertension prevalence compared to those in quartile 1 (0–75 μg/day), and a significant p-value for the trend through quartiles (0.0267). In addition, we used smooth curve fitting to demonstrate a persistent negative linear association between dietary selenium intake and the prevalence of hypertension (**Figure 2**), consistent with the results shown in **Table 2**.

**Table 2:** Association of with dietary selenium intake and prevalence of hypertension.

	Model 1		Model 2		Model 3	
	OR 95% CI	p-Value	OR 95% CI	p-Value	OR 95% CI	p-Value
Dietary selenium intake(100 μg/day)	0.78 (0.74, 0.81)	<0.0001	0.97 (0.92, 1.03)	0.3202	0.89 (0.80, 1.00)	0.0425
Stratified by Dietary selenium intake(100 μg/day) quartiles						
Q 1 (0 – 0.75)	Ref.		Ref.		Ref.	
Q 2 (0.76 – 1.03)	0.90 (0.84, 0.96)	0.0009	0.91 (0.86, 1.00)	0.0398	0.81 (0.69, 0.94)	0.0074
Q 3 (1.04 – 1.38)	0.84 (0.79, 0.89)	<0.0001	0.95 (0.89, 1.03)	0.2086	0.82 (0.70, 0.95)	0.0107
Q 4 (1.39 – 4.00)	0.71 (0.66, 0.75)	<0.0001	0.95 (0.88, 1.02)	0.0254	0.80 (0.68, 0.94)	0.0084
*p* for trend	<0.0001		0.3222		0.0267	

In sensitivity analysis, dietary selenium intake was converted from a continuous variable to a categorical variable (quartiles). OR, odds ratio; 95% CI, 95% confidence interval. Model 1: No covariates were adjusted. Model 2: Adjusted for gender, age, and race. Model 3: Adjusted for gender, age, race, education, PIR, BMI, WC, alcohol, smoking, diabetes, HbA1c, Scr, UC, TC, TG, HDL-C, LDL-C, HB, ALB, ALT, AST, SBP and DBP.

### Subgroup analysis and interaction test

**Figure 3:**
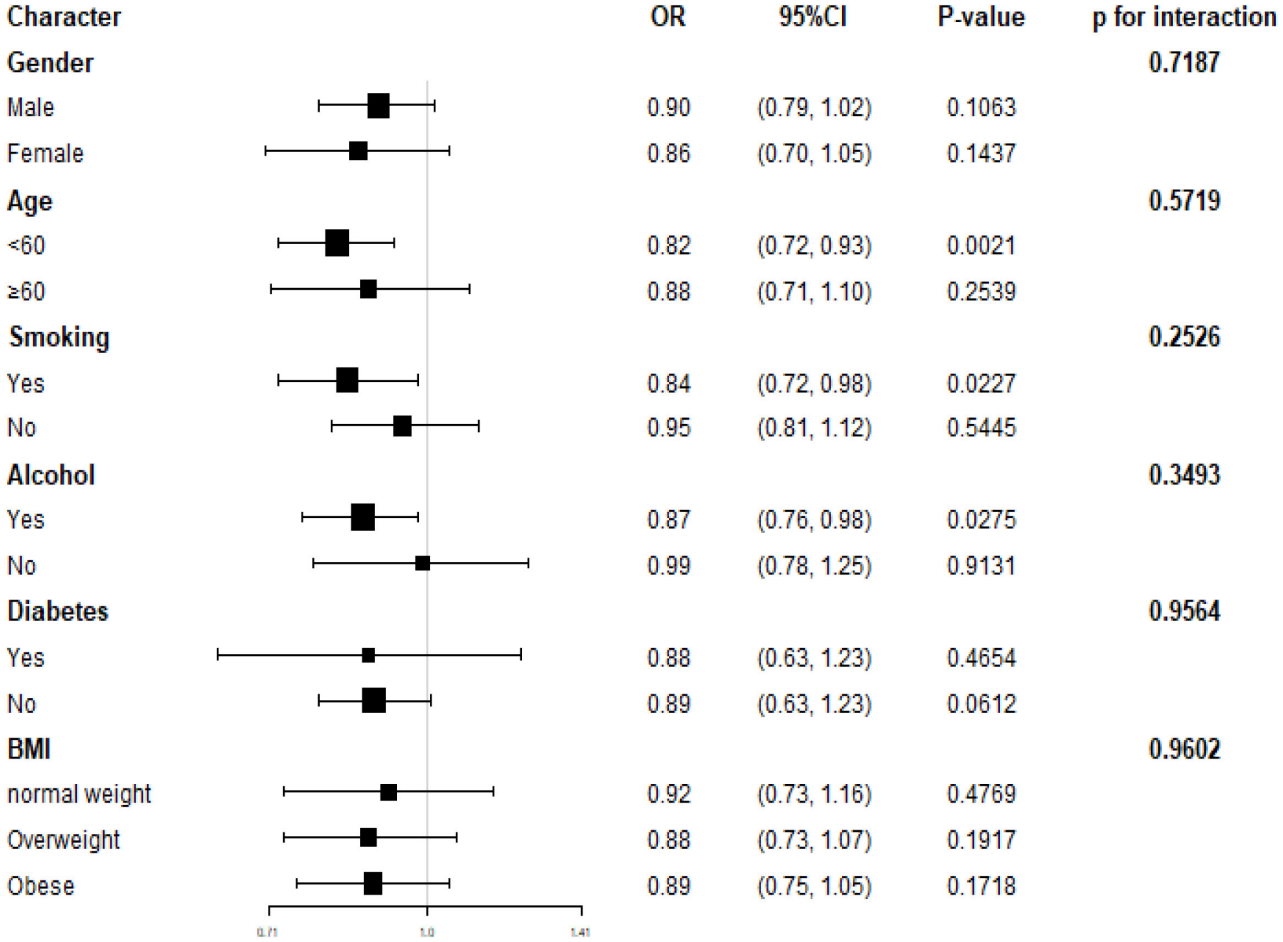
Subgroup analyses for the relationship between dietary selenium intake and hypertension.

We conducted subgroup analyses to examine the relationship between dietary selenium intake and hypertension prevalence (refer to **Figure 3**). Our findings suggest that reduced dietary selenium intake is not consistently associated with a higher prevalence of hypertension in certain subgroups. Specifically, this correlation was not statistically significant (P>0.05) for all genders, participants with diabetes, older individuals, non-alcohol consumers, and non-smokers. We also explored potential interactions with gender, age, BMI, smoking, alcohol consumption, and diabetes (**Figure 3**), but none of these interactions were found to be statistically significant (all p-values for interaction > 0.05). This indicates that the association between dietary selenium intake and hypertension prevalence is not influenced by these factors. Our results suggest that the inverse association between dietary selenium intake and hypertension prevalence is consistent across different genders, age groups, BMI categories, smoking and alcohol statuses, as well as diabetes statuses, and may be relevant in various population settings.

## Discussion

The study involving 32,928 adults found a significant inverse correlation between dietary selenium intake and hypertension. This suggests that individuals with higher dietary selenium intake may have a lower prevalence of hypertension. Interestingly, this association remained consistent across different demographic groups, including age, gender, BMI, smoking and alcohol use statuses, and diabetes statuses. These findings highlight the potential benefits of increasing dietary selenium intake for reducing the prevalence of hypertension in various populations.

Selenium is a vital micronutrient that is crucial for maintaining human health, particularly in the areas of energy metabolism and gene expression (7). Additionally, selenium performs a range of important functions, such as acting as an antioxidant (8), reducing inflammation (9), regulating the immune system (10), promoting anti-aging effects (11), and potentially inhibiting tumor growth (12). One of the most prevalent and extensively distributed members of the glutathione peroxidase (GPx) class of selenium-dependent enzymes, GPx-1 is responsible for ensuring that cells have the proper amounts of reduced glutathione. Reduced glutathione helps to scavenge dangerous chemicals, such as hydrogen peroxide and lipid hydroperoxides, and prevents them from accumulating within the cell, so protecting the cell from oxidative damage. Selenium deficiency has been associated with various chronic metabolic diseases, including hyperlipidemia, atherosclerosis, hyperglycemia, and hyperphenylalaninemia (13). While micronutrient supplements are becoming increasingly popular for improving health, excessive selenium intake can be harmful. The recommended daily intake for adults is 70μg, with a tolerable upper limit of 400μg. Previous research has indicated a link between dietary selenium intake and various clinical conditions. For instance, high selenium intake has been associated with an increased risk of osteoarthritis (OA), while lower intake (less than 100μg) is not associated with an increased risk of OA (14). It has been found that excessive selenium intake can disrupt the insulin signaling pathway, leading to hyperglycemia and obesity, contributing to systemic inflammation, which is a significant factor in OA and hypertension (15). Furthermore, dietary selenium intake was found to be negatively associated with kidney stones (16), post-stroke depression (17), and stroke risk in adults over the age of 60 (18).

Hypertension is a common cardiovascular disease characterized by a sustained elevation of blood pressure, which can lead to alterations and damage to the structure and function of the heart, brain, kidneys, and fundus. Therefore, the management and prevention of hypertension have become a global public health priority (19). Previous studies have shown mixed results regarding the relationship between blood selenium levels and hypertension. For example, the Kuopio Ischemic Heart Disease Risk Factor Study found a negative correlation between serum selenium and systolic blood pressure (20), while a study of elderly men in rural Finland found no correlation (21). Additionally, a cross-sectional study of 2,638 individuals aged 40 and above in the NHANES revealed a link between high serum selenium concentrations and a higher incidence of hypertension (22), suggesting the need for further research in this area.

Despite being a widespread chronic illness, hypertension’s precise molecular causes are unknown, despite the fact that a number of studies have demonstrated a significant role for inflammation in the onset and progression of the condition (23). The association between inflammatory indicators and hypertension risk was evaluated by investigators. through a meta-analysis of cohort studies. They discovered that elevated levels of hs-CRP and IL-6 were linked to a higher risk of hypertension (24). A more recent study by Mohammad Gholizade et al. found that selenium supplementation reduced CRP and IL-6 plasma concentrations (25). The production of interleukin-6 is strongly correlated with the activation of the nuclear factor κ b (NF - κ b) signaling pathway, which is linked to heightened inflammatory responses. Because selenium controls the expression of the selenoprotein gene, it may prevent NF-kappaB activation. Furthermore, selenium supplementation in chronic inflammation may lessen the inflammatory process by boosting the synthesis of selenium-containing proteins to replenish the liver’s and the blood’s depleted selenium stores, which will stop the generation of CRP (26). It has been demonstrated that arterial stiffness is a significant cause of Isolated systolic hypertension in the elderly (27), increased risk of stroke (28), coronary artery disease, and heart failure that are associated (29). Arterial stiffness is associated with increased angiotensin II activity, increasing NADPH oxidase activity, decreasing NO bioavailability, and increasing reactive oxygen species production (30, 31). Hypertension is commonly distinguished by endothelial dysfunction, a condition predominantly characterized by a diminished bioavailability of NO. Endothelial NO synthase (eNOS) is essential for NO production in endothelial cells; inhibition or dysfunction of eNOS results in NO deprivation, which hinders endothelium-dependent vasodilation and exacerbates the progression of hypertension. On the contrary, selenium increases NO synthesis by stimulating NO synthase (NOS) activity (32). Selenium additionally inhibits NO degradation and preserves NO stability (33). Arterial stiffness primarily arises from vascular remodeling, characterized by alterations in the structure and function of blood vessel walls. These modifications include wall thickening, an elevation in the wall-to-lumen ratio, and a reduction in microarterioles (34). These modifications result in atypical vascular functioning, impacting circulation, oxygenation, and blood provision (35). Furthermore, inflammatory factors, including IL-1, IL-17, and IL-6, are stimulated by AngII signaling (27). Arterial rigidity is associated with inflammation; vascular remodeling is slowed by inhibiting inflammation, and the risk of hypertension is decreased (36).

Our study has strengths, including a focus on dietary selenium intake and its association with hypertension, which is a novel contribution compared to previous research that primarily concentrated on serum selenium levels. Additionally, we rigorously adjusted for confounding variables to enhance the reliability of our findings. Nevertheless, it is crucial to recognize the shortcomings of our study. To begin with, the cross-sectional design constrains our capacity to establish causation, allowing only for the identification of correlations. In addition, the reliance on self-reported dietary information from the NHANES questionnaire introduces potential subjective bias. Moreover, the questionnaire may not accurately capture long-term dietary patterns due to the variability in an individual’s daily diet. Lastly, genetic variation, dietary habits, and socioeconomic factors are also significant contributors to cardiovascular disease susceptibility. Therefore, more research and research is needed on whether the conclusions drawn in this study apply to populations of different ethnicities and regions.

## Conclusion

Our findings suggest an independent association between higher dietary selenium intake and reduced hypertension prevalence, warranting further large-scale prospective studies for validation.

## Data availability statement

The original contributions presented in the study are included in the article/supplementary material. Further inquiries can be directed to the corresponding author.

## Ethics statement

This study was approved by the National Center for Health Statistics Research Ethics Review Board. The participants provided informed consent to participate in the NHANES survey. The NHANES protocol complies with the U.S. Department of Health and Human Services Policy for the Protection of Human Research Subjects. NCHS IRB/ERC Protocol number: 2011-17. Ethical review and approval were waived for this study as it solely used publicly available data for research and publication.

## Author contributions

YW: Writing – original draft. ZY: Writing – original draft, Writing – review & editing.
